# Crystal Structures of Large-Volume Commercial Pharmaceuticals

**DOI:** 10.1063/4.0000808

**Published:** 2025-10-27

**Authors:** James A Kaduk, Anja Dosen, Thomas N Blanton

**Affiliations:** 1 North Central College, Naperville, IL, USA; 2 ICDD, Newtown Square, PA, USA

## Abstract

As part of a continuing project, the room-temperature crystal structures of 17 commercial pharmaceutical APIs have been solved and refined using synchrotron X-ray powder diffraction data (11-BM at APS and Wiggler Low Energy Beamline at CLS), and optimized using density functional techniques. These include: delamanid C25H25F3N4O6 (Deltyba®), diroximel fumarate C11H13NO6 (Vumerity®), sparsentan C32H40N4O5S (Filspari®), trametinib dimethyl sulfoxide C26H23FIN5O4(C2H6OS) (Mekinist®), etrasimod C26H26F3NO3 (Velsipity®), anisomycin C14H19NO4, iprodione C13H13Cl2N3O3 (Rovral®), flumethasone C22H28F2O5, givinostat hydrochloride monohydrate Form I C24H28N3O4Cl(H2O) (Duvyzat™), aprocitentan Form A C16H14Br2N6O4S (Tryvio™), ethynodiol diacetate C24H32O4 (Ovulen), repotrectinib C18H18FN5O2 (Augtyro™), fruquintinib Form I C21H19N3O5 (Fruzaqla®), quizartinib hydrate C29H32N6O4S(H2O)1/3 (Vanflyta®), cabotegravir C19H17F2N3O5 (Vocabria), pirtobrutinib Forms 1 and 2 C22H21F4N5O3 (Jaypirca®), and protriptyline hydrochloride C19H22NCl (Vivactil®). Other new structures may be presented as they become available.

